# Reduced IQGAP2 expression promotes EMT and inhibits apoptosis by modulating the MEK-ERK and p38 signaling in breast cancer irrespective of ER status

**DOI:** 10.1038/s41419-021-03673-0

**Published:** 2021-04-12

**Authors:** Dinesh Kumar, Saket Awadesbhai Patel, Md. Khurshidul Hassan, Nachiketa Mohapatra, Niharika Pattanaik, Manjusha Dixit

**Affiliations:** 1 School of Biological Sciences, National Institute of Science Education and Research Bhubaneswar, HBNI, P.O. Jatni, Khurda, Odisha 752050 India; 2 Apollo Hospitals, Plot No. 251, Old Sainik School Road, Bhubaneswar, Odisha 750015 India; 3 AMRI Hospital, Plot No. 1, Near Jayadev Vatika Park, Khandagiri, Bhubaneswar, Odisha 751019 India

**Keywords:** Breast cancer, Cancer

## Abstract

IQGAP2, a member of the IQGAP family, functions as a tumor suppressor in most of the cancers. Unlike IQGAP1 and IQGAP3, which function as oncogenes in breast cancer, the role of IQGAP2 is still unexplored. Here we report a reduced expression of IQGAP2, which was associated with lymph node positivity, lymphovascular invasion, and higher age in breast cancer patients. We found an inverse correlation of IQGAP2 expression levels with oncogenic properties of breast cancer cell lines in estrogen receptor (ER) independent manner. IQGAP2 expression enhanced apoptosis via reactive oxygen species (ROS)-P38-p53 pathway and reduced epithelial–mesenchymal transition (EMT) in a MEK-ERK-dependent manner. IQGAP2-IQGAP1 ratio correlated negatively with phospho-ERK levels in breast cancer patients. Pull-down assay showed interaction of IQGAP1 and IQGAP2. IQGAP2 overexpression rescued, IQGAP1-mediated ERK activation, suggesting the possibility of IQGAP1 sequestration by IQGAP2. IQGAP2 depletion, in a tumor xenograft model, increased tumor volume, tumor weight, and phospho-ERK expression. Overall, our findings suggest that IQGAP2 is negatively associated with proliferative and metastatic abilities of breast cancer cells. Suppression of IQGAP1-mediated ERK activation is a possible route via which IQGAP2 restricts oncogenic properties of breast cancer cells. Our study highlights the candidature of IQGAP2 as a potent target for therapeutic intervention.

## Introduction

Breast cancer is the leading cause of cancer-related mortalities in women^1^. Although the survival rate of breast cancer patients has improved, yet long-term survival remains low^2^. Despite the fact that many genes have been identified^3^, identification of additional players, which can work independent of breast cancer molecular subtype, is needed. IQ motif containing GTPase activating proteins (IQGAPs) is a class of scaffolding proteins containing three members, namely IQGAP1, IQGAP2, and IQGAP3, which share high similarity at five domains^4^, yet show diverse cellular functions^5^. Increased IQGAP1 and IQGAP3 levels have been found in many cancers and promote tumor growth and metastasis^4,6–8^. While most of the studies have reported reduced expression of IQGAP2 in cancers^9–12^, yet, a couple of studies observed elevated IQGAP2 levels in cancers^13,14^. Multiple studies have highlighted the role of IQGAP1 as a potent oncogene, associated with worse prognosis in breast cancer^15–18^. The role of IQGAP2 on the other hand is still unexplored in breast cancer. Our own data mining study indicated a tumor-suppressive function for IQGAP2^19^. Interestingly, some studies highlighted the reciprocal expression pattern of IQGAP1/IQGAP2 in cancers, hinting at tight regulation of one isoform over another^11,20,21^.

We carried out this study with the primary aim to establish the role and molecular mechanism of IQGAP2 in breast cancer progression and its relation with IQGAP1. This study enabled us to deduce the role of IQGAP2 in predicting survivability of breast cancer patients via its role in regulating oncogenic properties of breast cancer cells, association with IQGAP1, and correlation with markers of invasion and metastasis, using in vivo, in vitro, and clinical specimens. The findings of this study will thus prove beneficial in evaluating the candidature of IQGAP2 as a biomarker and as a therapeutic target.

## Materials and methods

### Breast cancer sample collection

For this study a total of 226 tumor and 63 adjacent/normal formalin-fixed paraffin-embedded (FFPE) archival breast tissue samples were used. Out of these, 126 tumors and 53 adjacent normal tissue samples were collected from the SRL Diagnostic Lab and Department of Pathology, Apollo Hospitals, Bhubaneswar. Additionally, breast cancer tissue microarray (BC081120c) was purchased from BioMax (MD, USA). The study was approved by the Institutional Ethics Committee, NISER, Bhubaneswar (protocol no. NISER/IEC/2016-01). For each patient, we recorded histological type, tumor size, tumor–nodes–metastasis stage, lymphovascular invasion, and age.

### Immunohistochemistry

In total, 5 µm thick FFPE tissue sections were deparaffinized in xylene and rehydrated in a series of graded alcohol. Antigen retrieval was performed in a low pH (citrate, pH 6.8) buffer. Endogenous peroxidase activity was blocked by Envision Peroxidase Blocker (Dako, CA, USA). Tissue sections were incubated for 1 h with primary antibodies against IQGAP2, IQGAP1 (Abcam, MA, USA, 1:100 dilution), and phospho-ERK (CST, MA, USA, 1:100 dilution) followed by incubation with Envision Flex horseradish peroxidase (HRP), HRP secondary antibody (Dako) for 30 min. Color development was done using liquid DAB substrate and counterstained with hematoxylin. The scoring of IQGAP2, IQGAP1, and phospho-ERK immunohistochemistry (IHC) data was done according to the Allred scoring system^22^.

### Cell culture, plasmids, and stable line preparation

MCF7 and MDA-MB-468 cells were purchased from NCCS, Pune, India and cultured at 37 °C, 5% CO_2_ in DMEM and RPMI 1640 (HiMedia, Mumbai, India) supplemented with 10% fetal bovine serum (US origin, HiMedia), respectively. For transient expression or knockdown of IQGAP2, 1 × 10^6^ cells were transfected with pCMV6_IQGAP2_myc (Origene, MD, USA) or pLKO.1_IQGAP2_shRNA vectors (Sigma, Missouri, USA) using Lipofectamine 3000 (Thermo Scientific, MA, USA). For stable expression, cells were grown in complete media supplemented with G418 (1000 µg/ml) or puromycin (1 µg/ml) antibiotics, respectively.

### Assays for tumorigenic properties of cells and expression analysis

Detailed methodology is given in Supplementary methods.

### Cell apoptosis assay

Apoptosis was measured using 1 × 10^6^ cells in a 6-well plate by FITC Annexin V Apoptosis Detection Kit I (BD Pharmingen™, NJ, USA) as per manufacturer’s instruction. The cells were analyzed using FACS Calibur (BD Biosciences, CA, USA) flow cytometry. Data were analyzed using cell quest pro software (BD Biosciences).

### Measurement of caspase activity

Caspase activity in cells was measured using the Caspase-Glo 3/7 Assay Kit (Promega) in 1 × 10^4^ cells, as per manufacturer’s instructions. Luminescence was measured in a Varioscan Flash (Thermo Scientific) luminometer.

### Measurement of reactive oxygen level

In total, 2 × 10^4^ cells were plated in a 96-well plate using phenol red free medium. Cells were stained with diluted DCFDA solution as per the manufacturer’s protocol (DCFDA/H2DCFDA Cellular ROS Assay Kit, Abcam). Images were captured at ×4 magnification using the FITC filter. The intensity of fluorescence was analyzed using ImageJ software.

### Phospho-ERK and phospho-P38 modulation

In total, 0.4 × 10^6^ cells were plated in a 12-well plate and cultured for 24 h. Cells were washed and treated with ERK inhibitor for 30 min (10 µM, ERK inhibitor II, U0126, Sigma), with p38 inhibitor for 2 h (1 µM, SB202190, Sigma), and with p38 activator for 2 h (0.5 ng/ml, Anisomycin, TCI, Tokyo, Japan) along with DMSO control.

### Estrogen receptor activity and its inhibition

Cells were incubated in phenol red free growth medium. Western blot analysis and RT-PCR were used to estimate activated estrogen receptor (ER) and transcript levels. To inhibit ER, cells were treated with 1 µM Tamoxifen (T5648, Sigma) for 30 min^23^.

### Xenograft tumor growth

All animal experiments were approved by the Institutional Animal Ethics Committee, NISER, India. Female athymic nude mice (aged 6–8 week, 18–20 g) were purchased from the Central Animal facility, CCMB, Hyderabad, India. The mice were injected with 2 × 10^6^ cells into the mammary fat pad. After 30 days, the mice were sacrificed and the xenograft tumors were removed, weighed, and photographed.

### c-BioPortal database analysis

c-BioPortal site was used to visualize the protein mass-spectrometry (74 patients) data of Breast Invasive Carcinoma, TCGA (Firehose Legacy) dataset for IQGAP2, IQGAP1, and phospho-ERK for expression correlation analysis.

### Pull-down assay

Cell lysates were prepared by sonication in lysis buffer containing phosphatase-protease inhibitor (Thermo Scientific). Glutathione-sepharose beads were precleared and incubated with the equal amount of lysates from glutathione s-transferase (GST) tagged IQGAP1 or GST alone. Western blotting for IQGAP2, IQGAP1, and GST was used for detection.

### Statistical analysis

GraphPad Prism 6.0 Version (GraphPad Software Inc., CA, USA) and Microsoft excel (Microsoft, Washington, USA) were used for all statistical analyses. Continuous data were shown as mean ± standard error of mean and analyzed by Student’s *t* test (two-tailed, unpaired). Mann–Whitney *U* test was utilized to determine the significance of difference in distribution frequency of Allred scores. Correlations were calculated by Pearson correlation. Chi-square test and odds ratio were used in analysis of data in 2 × 2 contingency tables. *p* ≤ 0.05 was considered to be significant for all the tests.

## Results

### Reduced expression of IQGAP2 in breast cancer tissues

**Fig. 1:**
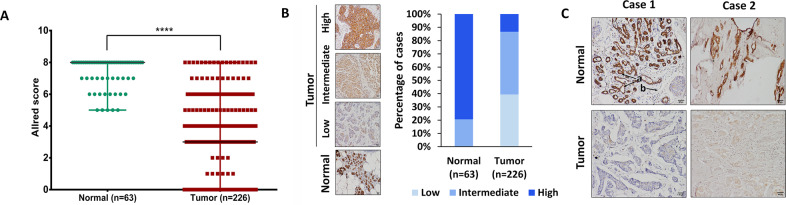
Reduced expression of IQGAP2 in breast cancer tissues. **A** The comparison of Allred scores of IQGAP2 expression between tumor (*n* = 226) and adjacent normal tissue (*n* = 63) of breast cancer patients. The Allred score for IQGAP2 is significantly (Mann–Whitney *U* test) reduced in tumor tissues (median = 3) compared to normal (median = 8). **B** Representative images and distribution frequency of normal versus cancer tissues according to the Allred score of IQGAP2. For statistical analyses, the Allred scores of 0–2 were treated as negative or weak, 3–6 as moderate, and 7–8 as strong expression levels. *y*-axis represents the percentage of patients positive with IQGAP2 low, intermediate, or high Allred score. *x*-axis represents two groups; normal and tumor. **C** The representative images of tumor tissue and adjacent normal tissue of two breast cancer patients, showing IQGAP2 expression and localization. **C**-a Indicates the glandular cells and **C**-b shows the stromal region. The images were captured using a ×10 objective lens of bright field microscope. Scale bar in all images is 50 microns, AS Allred score, ****p ≤ 0.0001.

To identify the expression level of IQGAP2 in breast cancer, we performed IHC in breast cancer patients. We found reduced expression of IQGAP2 in tumor tissue compared to normal tissue (Fig. 1A). Most of the breast cancer patients showed low expression in tumor tissue and moderate or strong expression in adjacent normal tissue (Fig. 1B). Analysis of cell-type-specific expression revealed higher expression of IQGAP2 specifically in glandular cells than the stromal cells (Fig. 1C a, b). IQGAP2 expression was predominant in the cytosolic region, and there was no difference in the localization pattern between normal or tumor tissues (Fig. 1C).

### Low expression of IQGAP2 associates with poor clinical outcomes

We divided patients into two groups, IQGAP2 low and IQGAP2 high, based on IQGAP2 IHC scores and compared the frequency of clinicopathological characteristics between them. Analysis showed that lower IQGAP2 expression in breast cancer was significantly associated with higher age, lymph node metastasis, lymphovascular invasion, and higher cancer stage, but not with tumor size (Table 1).

**Table 1:** Correlation of IQGAP2 expression with histopathological parameters of breast cancer.

Characteristics	No. of cases	IQGAP2 expression		*p* value^a^
		Low (%)	High (%)	
Age (years)				
≤40	44	32 (72.73)	12 (27.27)	**0.002**
>40	182	164 (90.1)	18 (9.89)	
Tumor size (cm)				
≤5	125	107 (85.6)	18 (14.4)	0.61
>5	99	87 (87.88)	12 (12.12)	
Lymph node metastasis				
N0	109	91 (83.48)	18 (16.51)	
N1	48	39 (81.25)	9 (18.75)	0.81^b^
N2	63	60 (95.23)	3 (4.76)	**0.02**^c^
Lymphovascular invasion				
Yes	61	58 (95.08)	3 (4.92)	**0.0017**
No	64	48 (75)	16 (25)	
Cancer stage				
I–II	116	93 (80.17)	23 (19.83)	**0.00013**
III–IV	101	98 (97.03)	3 (2.97)	

^a^Chi-square goodness of fit test.

^b^N0 versus N1.

^c^N0 versus N2.

Bold values show statistically significant association.

### IQGAP2 expression does not correlate with breast cancer molecular subtype

To determine the correlation of molecular subtypes of breast cancer with IQGAP2 expression levels, we determined mRNA and protein expression levels in cells of different breast cancer molecular subtypes. We observed very low expression of IQGAP2 in T-47D, MCF 10A, and MDA-MB-231, at protein (Supplementary Fig. 1A) and mRNA (Supplementary Fig. 1B) levels. On the other hand, MCF7, MDA-MB-453, and MDA-MB-468 cell lines showed higher expression, showing no clustering of specific molecular subtype, based on IQGAP2 expression level. Similarly, other two members of the IQGAP family (IQGAP1 and IQGAP3) although showed expression in all the cell lines with some variation, yet there was no correlation with the molecular subtype (Supplementary Fig. 1A, B). Immunocytochemistry analysis showed no difference in localization pattern of IQGAP2 between MCF7 (ER/PR positive) and MDA-MB-468 (ER/PR/Her2 negative) cell lines (predominantly cytoplasmic in both) (Supplementary Fig. 1C, D).

### IQGAP2 expression affects tumorigenic properties of breast cancer cell lines irrespective of ER status

**Fig. 2:**
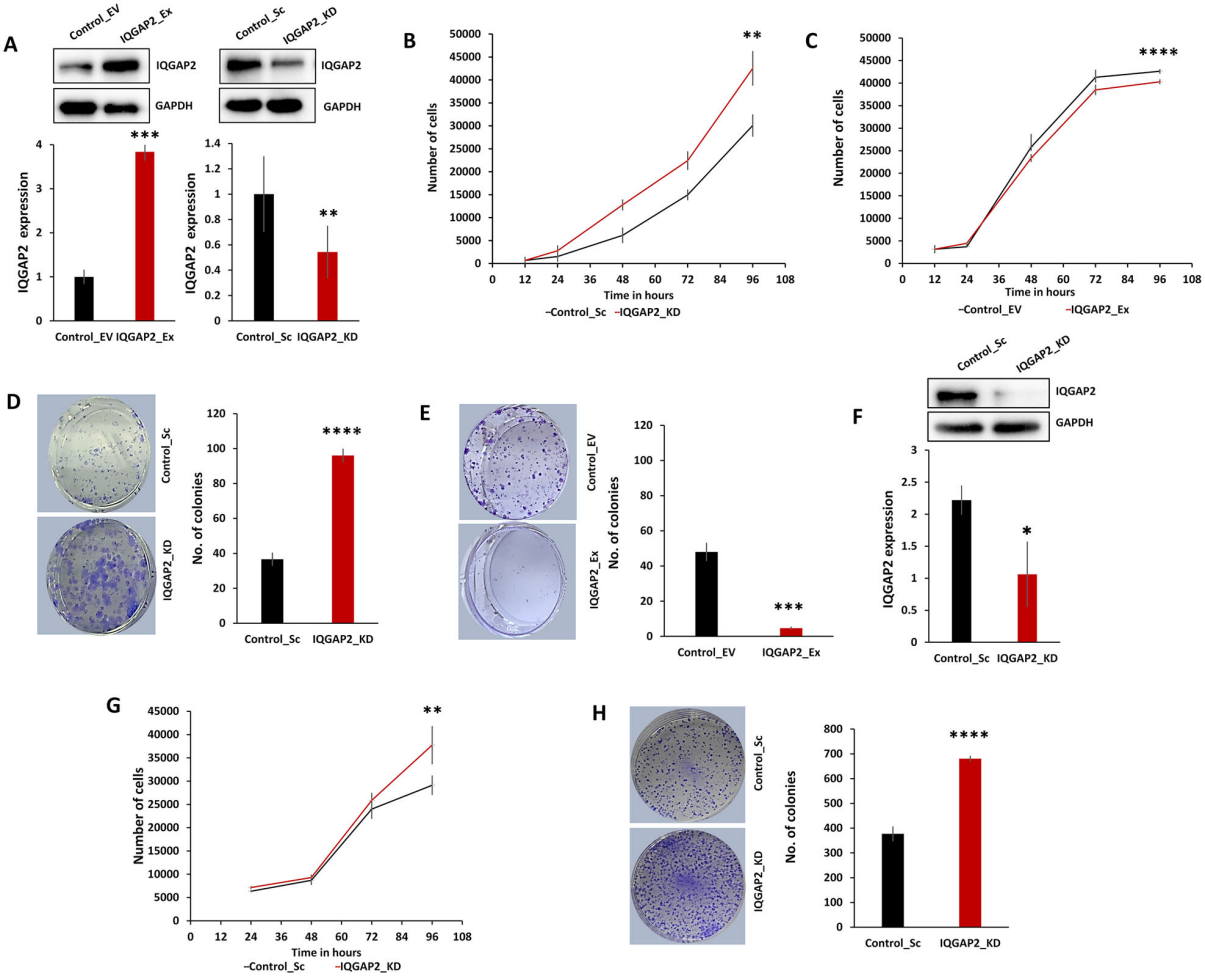
Reduced IQGAP2 expression promotes cell proliferation in breast cancer cell lines independent of ER status. **A** The upper panels show representative Western blot images of IQGAP2 expression in stable MCF7_Control_EV, MCF7_IQGAP2_Ex, MCF7_Control_Sc, and MCF7_IQGAP2_KD, respectively. Lower panels showing the relative densitometry bar graph of respective images. **B**, **C** The graph shows cell proliferation (MTS assay) of MCF7 IQGAP2_KD and MCF7 IQGAP2_Ex, respectively, at different time intervals. **B** Knockdown of IQGAP2 shows significant increase in cell proliferation. **C** Overexpression of IQGAP2 shows significant decrease in cell proliferation. **D** Left panel shows the images of colony formation assay in MCF7 IQGAP2_KD. Representative bar graph in the right panel showing a significantly higher number of colonies in the IQGAP2 knockdown group than in the control group. **E** Image showing the number of colonies in MCF7 IQGAP2_Ex group and MCF7 Control_EV group (left panel). The right panel bar graph showing the differences of colony numbers between both the groups. Overexpression of IQGAP2 shows significantly low colony number compared to control. **F** Western blot image showing (upper panel) knockdown of IQGAP2 in MDA-MB-468 cell lines and its relative densitometry bar graph (lower panels). **G** Bar graph shows the comparison between cell number (MTS assay) in MDA-MB-468 IQGAP2_KD group and Control_Sc group, from 24 to 96 h of cell plating. Knockdown of IQGAP2 shows higher cell proliferation rate than control. **H** Representative images of colony formation assay (left panel) in MDA-MB-468 IQGAP2_KD group and Control_Sc group. In the right panel, the bar graph shows the difference of colony numbers between both the groups. Knockdown of IQGAP2 shows significantly more number of colonies. Experiments were performed in triplicate and data were presented as mean ± SEM. Student’s *t* test, two-tailed unpaired was used for the comparison of means. *p ≤ 0.05, **p ≤ 0.01, ***p ≤ 0.001, ****p ≤ 0.0001.

We altered IQGAP2 expression stably in ER-positive cell line, MCF7 and a triple negative cell line, MDA-MB-468 (Fig. 2A, F). MTS (3-(4,5-dimethylthiazol-2-yl)-5-(3-carboxymethoxyphenyl)-2-(4-sulfophenyl)-2H-tetrazolium) and colony formation assays showed that in MCF7 cells, silencing of IQGAP2 increased cell proliferation (Fig. 2B, D), and ectopic expression of IQGAP2 reduced it (Fig. 2C, E). In the MDA-MB-468 cell line also IQGAP2 silencing led to a significant increase in cell proliferation (Fig. 2G), which was also supported by colony formation assay (Fig. 2H). Above results show that IQGAP2 inhibits cell proliferation irrespective of ER status of cell lines.

### IQGAP2 expression levels show dual effect on EMT and apoptosis

**Fig. 3:**
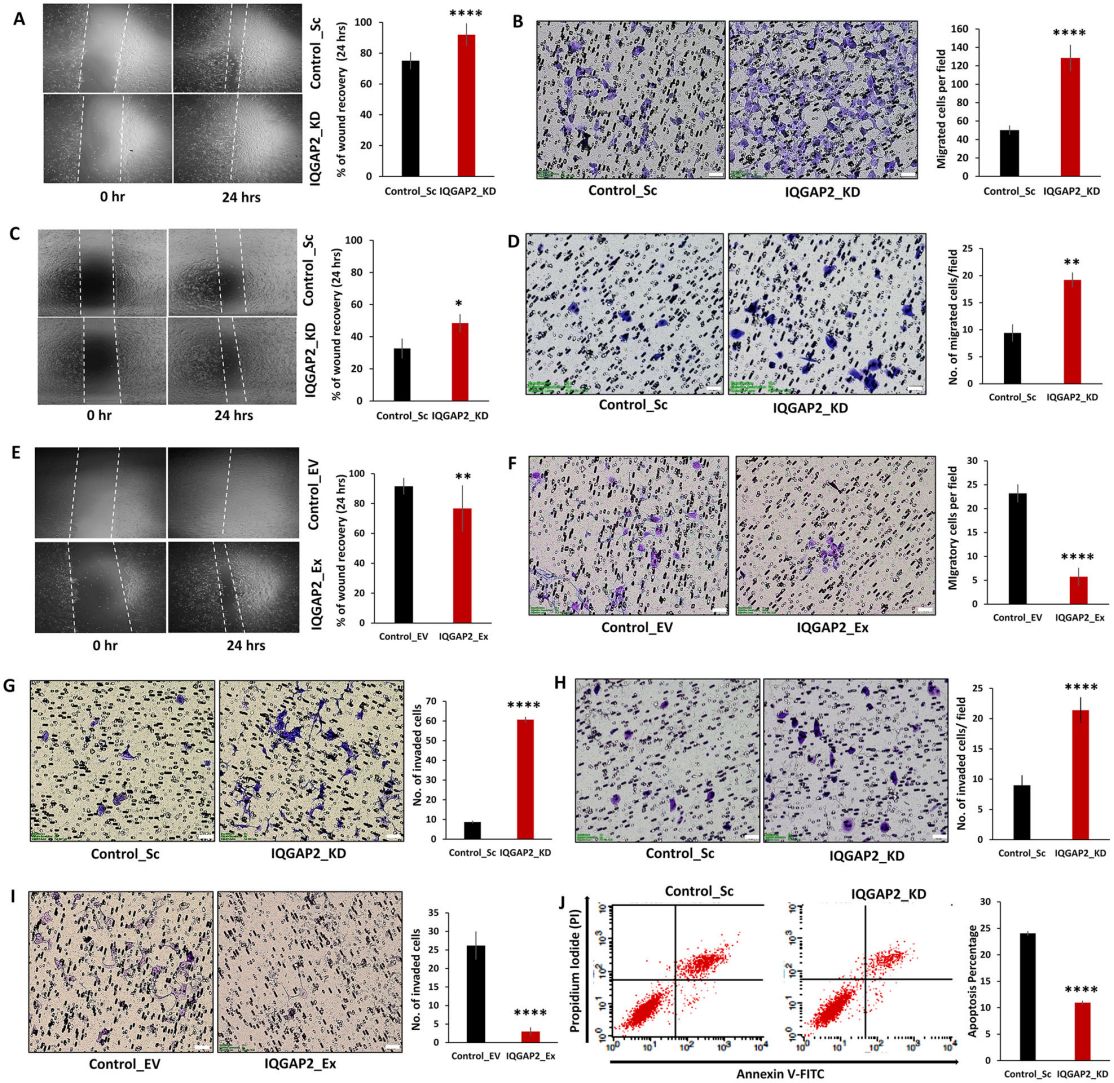
IQGAP2 expression levels show dual effect on EMT and apoptosis in opposite manner. **A** Wound healing assay in MCF7 IQGAP2_KD and MCF7 Control_Sc group showing higher recovery rate of wound in IQGAP2 knockdown group, compared to control. **B** Transwell migration assay in MCF7 IQGAP2_KD and MCF7 Control_Sc groups. Cells with IQGAP2 knockdown show enhanced transwell migration, compared to control. **C** Wound healing assay of MDA-MB-468 IQGAP2_KD and Control_Sc cells showing higher wound recovery rate of IQGAP2 knockdown group, compared to empty vector control. **D** Transwell migration assay in MDA-MB-468 IQGAP2_KD and Control_Sc groups shows enhanced transwell migration with IQGAP2 knockdown, compared to control. **E** Wound healing assay of MCF7 IQGAP2_Ex and MCF7 Control_EV cells showing reduced wound recovery with IQGAP2 overexpression, compared to control. **F** MCF7 cells with ectopic IQGAP2 expression (IQGAP2_Ex) show reduced transwell migration, compared to control (Control_EV). **G** MCF7 cells with IQGAP2 knockdown (IQGAP2_KD) show increased transwell invasion, compared to control (Control_Sc). **H** MDA-MB-468 cells with IQGAP2 knockdown (IQGAP2_KD) demonstrate enhanced transwell invasion, compared to control (Control_Sc). **I** MCF7 cells with ectopic IQGAP2 expression (IQGAP2_Ex) showing reduced transwell invasion, compared to control (Control_EV). **J** Cell apoptosis was measured by flow cytometry using 488 nm excitation and 647 nm emission filters in the MDA-MB-468 IQGAP2_KD and Control_Sc groups. The *x*-axis represents the cells positive for Annexin V stain and *y*-axis shows positive cells for propidium-iodide (PI) stain. Experiments were performed in triplicate and data are presented as mean ± SEM. *p ≤ 0.05, **p ≤ 0.01, ***p ≤ 0.001, ****p ≤ 0.0001. Student’s *t* test, two-tailed unpaired was used for the comparison of means. Scale bar in all images is 50 microns.

EMT is the hallmark of metastasis that is characterized by increase in migratory and invasive property of cells. The depletion of IQGAP2 level in MCF7 cells showed an increased rate of wound healing (Fig. 3A) and transwell migration (Fig. 3B). Similar trend was observed in MDA-MB-468 (Fig. 3C, D). Parallelly, ectopic expression of IQGAP2 reduced the rate of wound recovery and cell migration in MCF7 (Fig. 3E, F).

We further examined IQGAP2’s effect on the invasive property. Depletion of IQGAP2 level significantly increased the invasiveness of MCF7 cells (Fig. 3G) and MDA-MB-468 cells (Fig. 3H), whereas an opposite trend was observed in MCF7 having ectopic expression of IQGAP2 (Fig. 3I).

Zoheir et al.^20^ observed that silencing of IQGAP1 in HepG2 cells induces early and late apoptosis with upregulation of IQGAP2. In this study, we also made coherent observations in which depletion of IQGAP2 showed reduction in cell apoptosis in MDA-MB-468 (Fig. 3J) and MCF7 cells (Supplementary Fig. 2A). In contrast, ectopic expression of IQGAP2 increased the cellular apoptosis in MCF7 cells (Supplementary Fig. 2B). These results together indicate that reduction in IQGAP2 expression increases EMT and reduces apoptosis, both providing protumorigenic properties to the cells.

### IQGAP2 affects apoptosis by affecting p38-p53 pathway, triggered by increase in ROS

**Fig. 4:**
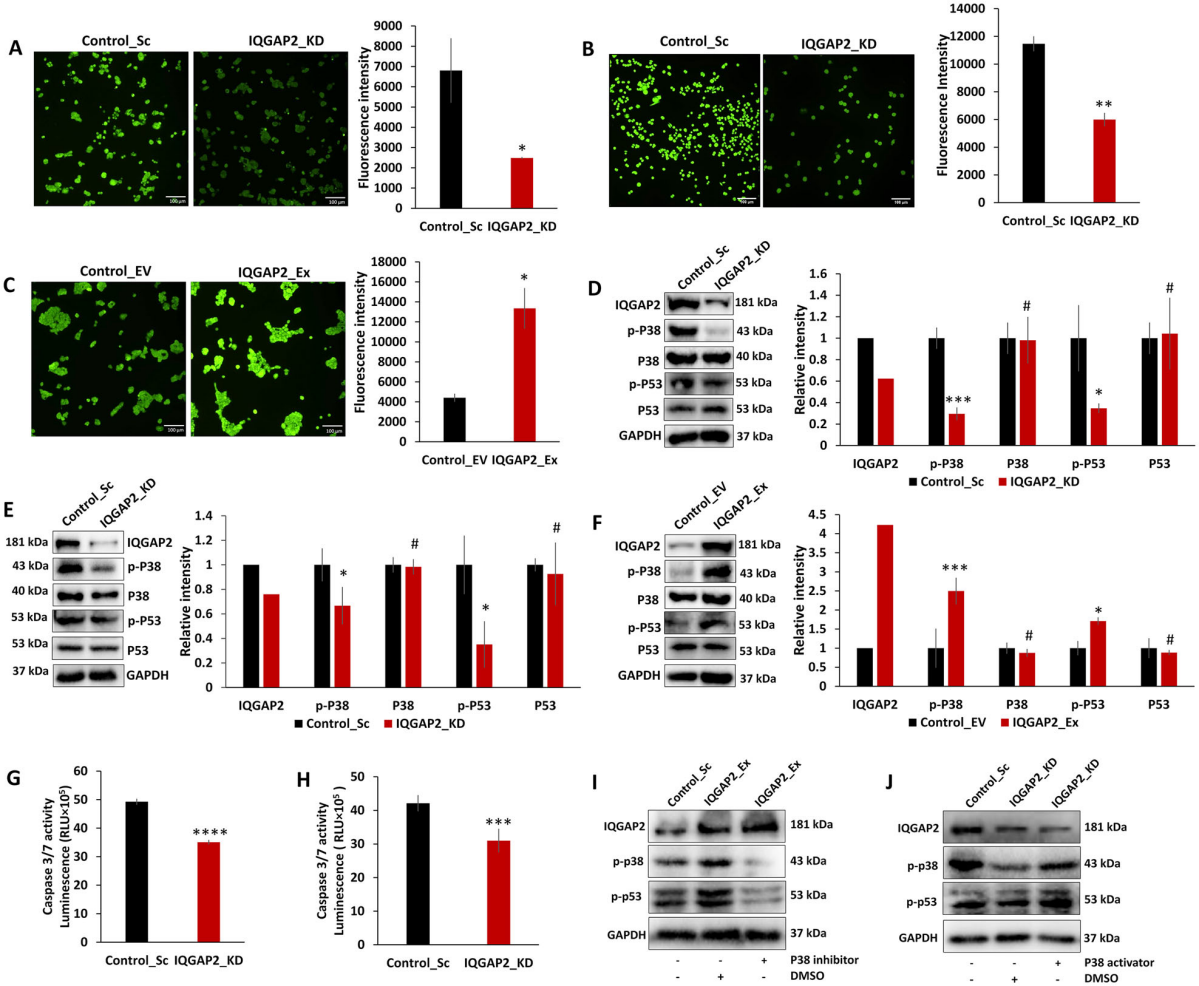
IQGAP2 modulates apoptosis by affecting the p38-p53 pathway triggered by increase in ROS. **A** IQGAP2 knockdown (IQGAP2_KD) in MCF7 shows reduction of ROS production, compared to control (Control_Sc). **B** IQGAP2 knockdown in MDA-MB-468 (IQGAP2_KD) shows reduction of ROS production, compared to control (Control_Sc). **C** IQGAP2 overexpression (IQGAP2_Ex) in MCF7 shows an increase of ROS production than control (Control_EV). **D** Representative images of Western blots of phospho-p38 and phospho-p53 in MCF7 with IQGAP2 depletion (IQGAP2_KD) and control (Control_Sc) groups (left panel). Graph showing reduced phospho-p53 and phospho-p38 level in IQGAP2 knockdown. **E** Representative images of Western blots of phospho-p38 and phospho-p53 in MDA-MB-468 with IQGAP2 depletion (IQGAP2_KD) and control (Control_Sc) group (left panel). Graph showing reduced phospho-p53 and phospho-p38 level in IQGAP2 knockdown (right panel). **F** Representative images of Western blots of phospho-p38 and phospho-p53 in MCF7 cells with IQGAP2 overexpression (IQGAP2_Ex) and control (Control_EV) group (left panel). Graph showing elevated phospho-p53 and phospho-p38 level in IQGAP2 overexpression group (right panel). **G** Graph showing caspase 3/7 levels in MCF7 with IQGAP2 depletion (IQGAP2_KD) and control (Control_Sc) groups. **H** Graph showing caspase 3/7 levels in IQGAP2 knockdown (IQGAP2_KD) in MDA-MB-468 and control (Control_Sc) groups. **I** Western blot images showing the expression level of phospho-p38 and phospho-p53 upon treatment with p38 inhibitor (SB202190, 1 µM) or vehicle control (DMSO) in MCF7_IQGAP2_Ex (IQGAP2 overexpression) group. **J** Western blot images showing the expression level of phospho-p38 and phospho-p53 upon treatment with p38 activator (anisomycin, 0.5 ng/ml) or vehicle control (DMSO) in MCF7_IQGAP2_KD (IQGAP2 depletion) group. Experiments were performed in triplicate and data are presented as mean ± SEM. ^#^*p* > 0.05, *p ≤ 0.05, **p ≤ 0.01, ***p ≤ 0.001, ****p ≤ 0.0001. Student’s *t* test, two-tailed unpaired was used for the comparison of means. Scale bar in all images is 100 microns.

Schmidt et al.^21^ observed an altered ROS level and apoptosis in IQGAP2−/− HCC mice. Parallelly we found that IQGAP2 depletion in MCF7 and MDA-MB-468 led to the reduced ROS generation (Fig. 4A, B), whereas the levels increased with IQGAP2 expression in MCF7 cells (Fig. 4C). The expression levels of ROS downstream targets, phospho-p38 MAPK and phospho-p53, were decreased with IQGAP2 depletion in MCF7 and MDA-MB-468 cells (Fig. 4D, E), and increased with IQGAP2 ectopic expression in MCF7 cells (Fig. 4F). IQGAP2 knockdown in MCF7 and MDA-MB-468 decreased the downstream effector caspase 3/7 level (Fig. 4G, H). To further authenticate, we found that IQGAP2 overexpression-induced elevated level of phospho-p38 and phospho-p53 was significantly inhibited by phospho-p38 inhibitor SB202190 (Fig. 4I). Similarly, IQGAP2 depletion-induced reduced level of phospho-p38 and phospho-p53 was rescued by phospho-p38 activator Anisomycin (Fig. 4J). These experiments confirm that IQGAP2 affects apoptosis via p38 and p53.

### IQGAP2 affects EMT via activation of ERK pathway

**Fig. 5:**
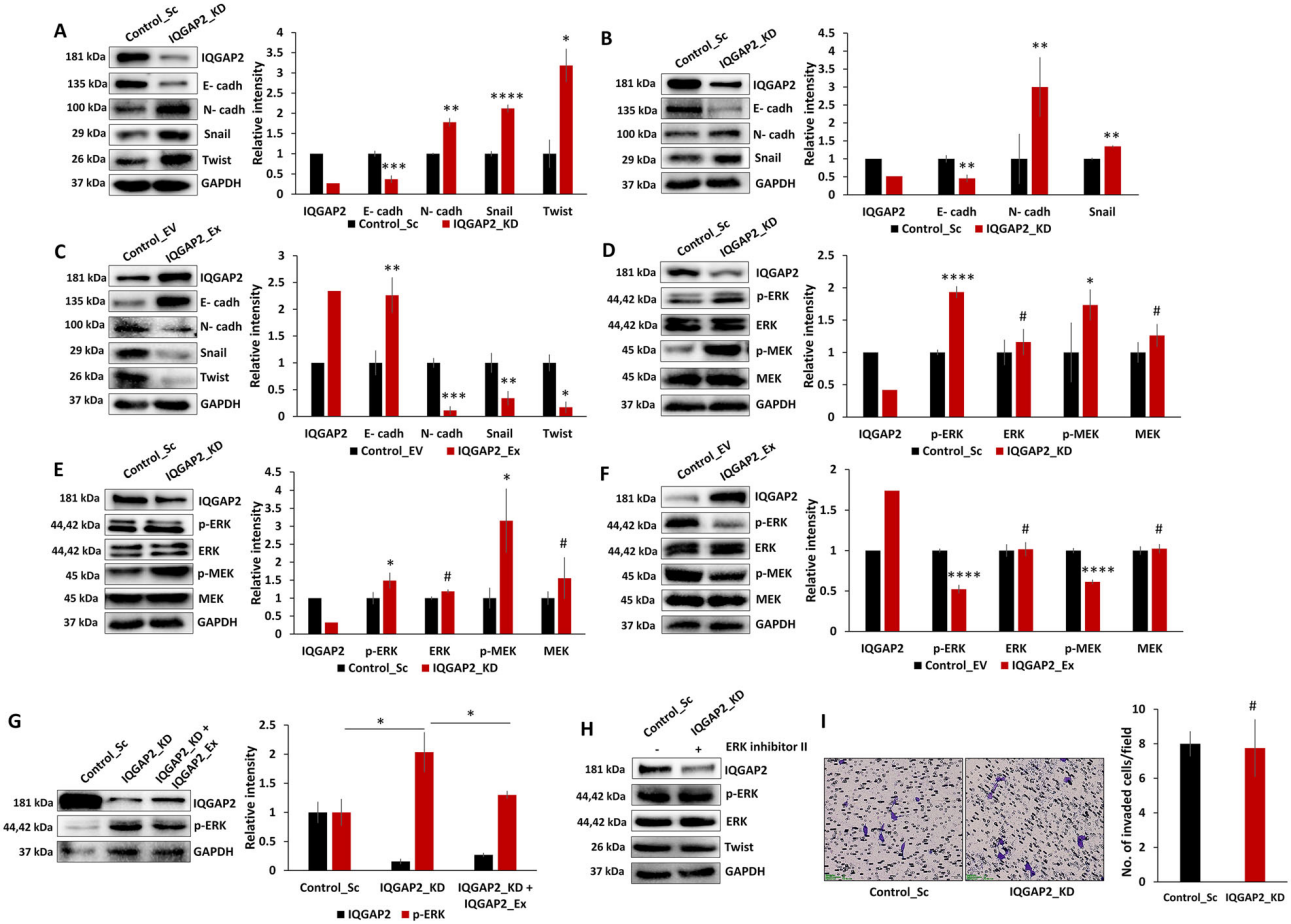
IQGAP2 affects EMT via activation of ERK pathway. Western blot showing elevated expression of N-cadherin, Snail, and Twist, and reduced level of E-cadherin in **A**, MCF7 with IQGAP2 depletion (IQGAP2_KD) compared to respective control (Control_Sc), and, in **B**, MDA-MB-468 with IQGAP2 depletion (IQGAP2_KD) compared to respective control (Control_Sc). **C** Western blot showing reduced expression of N-cadherin, Snail, and Twist and elevated level of E-cadherin in MCF7 with IQGAP2 overexpression (IQGAP2_Ex) compared to respective control (Control_EV). Western blot showing elevated expression of phospho-MEK and phospho-ERK in **D**, MCF7 with IQGAP2 depletion (IQGAP2_KD) compared to respective control (Control_Sc) and in **E**, MDA-MB-468 with IQGAP2 depletion (IQGAP2_KD) compared to respective control (Control_Sc). **F** Western blot showing reduced expression of phospho-MEK and phospho-ERK in MCF7 with IQGAP2 overexpression (IQGAP2_Ex) compared to respective control (Control_EV). **G** Western blot showing rescue of phospho-ERK in MCF7 with IQGAP2 depletion (IQGAP2_KD) upon IQGAP2 expression (IQGAP2_KD + IQGAP2_Ex). **H** Western blot showing rescue of Twist (phospho-ERK downstream target) after treating MCF7 with IQGAP2 depletion (IQGAP2_KD) with phospho-ERK inhibitor II. **I** Cell invasion assay after treating MCF7 with IQGAP2 depletion (IQGAP2_KD), using phospho-ERK inhibitor II (left panel). Right panel, bar graph showing no significant difference in no. of cells invaded in control (Control_Sc), and IQGAP2 depletion group (IQGAP2_KD) treated with phospho-ERK inhibitor II. Experiments were performed in triplicate and data are presented as mean ± SEM. ^#^*p* > 0.05, *p ≤ 0.05, **p ≤ 0.01, ***p ≤ 0.001, ****p ≤ 0.0001. Student’s *t* test, two-tailed unpaired was used for the comparison of means.

As IQGAP2 affects EMT, we checked its effect on EMT markers. MCF7 and MDA-MB-468 cells with IQGAP2 depletion showed lower levels of epithelial marker E-cadherin and higher levels of mesenchymal markers, N-cadherin, Snail, and Twist (Fig. 5A, B). Conversely, overexpression of IQGAP2 in MCF7 cells showed the opposite effect (Fig. 5C). Overall, these findings confirm that IQGAP2 regulates EMT in breast cancer cells.

Next, we checked MAPK/ERK and PI3K/AKT signaling pathways, which have been implicated in EMT in different cancer types. We found an elevated level of phospho-MEK and phospho-ERK1/2 with knockdown of IQGAP2 in MCF7 (Fig. 5D) and MDA-MB-468 (Fig. 5E). On the other hand, IQGAP2 overexpression decreased phospho-MEK and phospho-ERK1/2 in MCF7 (Fig. 5F). As further corroboration, ectopic IQGAP2 expression in MCF7-IQGAP2-KD cells showed rescue of the elevated phospho-ERK levels (Fig. 5G). Total AKT and its activated forms did not change in any experimental group (Supplementary Fig. 3A–C).

To further substantiate our findings, we found that IQGAP2 depletion-induced elevated level of phospho-ERK was significantly inhibited by ERK inhibitor U0126. The same experimental setup also showed rescue of Twist expression level (Fig. 5H) and abrogation of the invasiveness (Fig. 5I). These results confirm that reduced IQGAP2 expression induces EMT through the MEK/ERK pathway.

### Reduction in IQGAP2 activates ER in MCF7 cells through ERK

As IQGAP1 activates ERα^24^, we investigated the possibility of IQGAP2 suppressing phospho-ERα and ESR1 transcription. In MCF7 cells, IQGAP2 depletion increased ESR1 transcript level and vice versa (Supplementary Fig. 4A). In a similar way, IQGAP2 expression affected phospho-ERα level (Supplementary Fig. 4B, C).

We further investigated whether ER activation is downstream of ERK activation or otherwise. To check this, we first blocked ER activity with tamoxifen and analyzed the phospho-ERK, which did not show any significant change (Supplementary Fig. 4D). In contrast, inhibition of ERK resulted in reduction of phospho-ERα (Supplementary Fig. 4E), confirming that ER activation is ERK mediated. Depletion of IQGAP2 level significantly increased the amount of transcriptional targets of ERα, namely, progesterone receptor and pS2/trefoil factor 1, and vice versa (Supplementary Fig. 4F). The above results prove that IQGAP2 reduction activates ERα via ERK in MCF7 cells.

### Low IQGAP2 expression induces the pro-inflammatory cytokine expression in breast cancer cells

Inflammatory cytokines like CXCR1, IL-3, IL-5, IL-6, IL-8, IL-9, IL-10, CCL2, CCL3, and CCL11 have indispensable function in tumor initiation, progression as well as in metastasis^25^. We found a significant decrease in IL-6, IL-8, and CCL2 in MCF7_IQGAP2_Ex group (Supplementary Fig. 5A). In contrast, depletion of IQGAP2 level in MDA-MB-468 led to increased transcript levels of IL-6, CCL2, CCL3, and CCL11 (Supplementary Fig. 5B). Our results show coherence with the previous reports^26–29^ and explain how IQGAP2 might be affecting key cellular processes like proliferation and invasion through cytokines.

### Reduced expression of IQGAP2 promotes tumor growth in mouse model

**Fig. 6:**
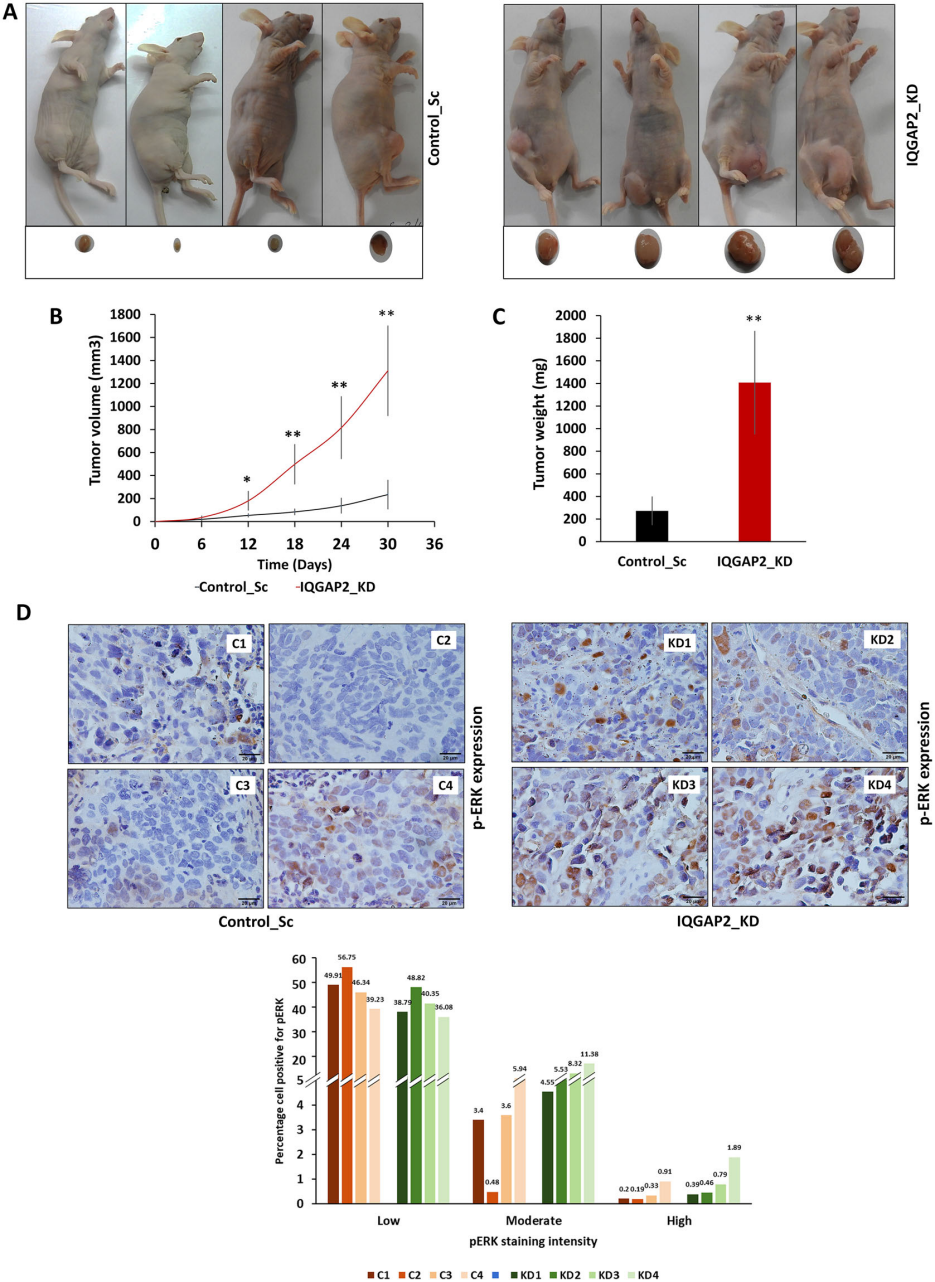
Reduced expression of IQGAP2 promotes tumor growth in mouse model. Two groups of nude mice were injected with MCF7 cells having IQGAP2 knockdown (IQGAP2_KD) or its control vector (Control_Sc). **A** Shows control (Control_Sc) and IQGAP2 knockdown (IQGAP2_KD) groups of nude mice and their tumors (*n* = 4). **B** Tumor volume of IQGAP2 knockdown (IQGAP2_KD) group and control (Control_Sc) group at the interval of 6 days (*n* = 4). Tumor volume was calculated using equation: volume = length × width^2^. **C** Tumor weight of IQGAP2 knockdown (IQGAP2_KD) and control (Control_Sc) mice groups at day 30 (*n* = 4). **D** Representative images of immunohistochemical staining for phospho-ERK in sections of control (C1, C2, C3, and C4 derived from MCF7_Control_Sc) and IQGAP2 knockdown (KD1, KD2, KD3, and KD4 derived from MCF7_IQGAP2_KD) nude mice xenograft-derived tumors (upper panel). Images were taken at ×40 with an upright bright field microscope. Graphs (lower panel) showing percentage of cells positive for low, moderate, or high phospho-ERK expression in xenografts derived from MCF7 with IQGAP2 knockdown (IQGAP2_KD) and its control (Control_Sc) from nude mice. The expression intensity was calculated using IHC profiler tool of ImageJ. *Y*-axis represents the percentage cell positivity for low, weak, or high phospho-ERK expression. *X*-axis shows mice in each group. Data are presented as mean ± SEM. Student’s *t* test, two-tailed unpaired was used for the comparison of means. *p ≤ 0.05, **p ≤ 0.01. The scale bar is 20 micron.

Further, we tested the effect of IQGAP2 on tumor growth in vivo, using a tumor xenograft model. IQGAP2 knockdown significantly increased both tumor volume and weight (Fig. 6A–C). Moreover, we found that the phospho-ERK expression in the IQGAP2 knockdown group was much higher compared to the control (Fig. 6D). This observation was in parallel with our in vitro data.

### IQGAP2 is negatively correlated with phospho-ERK and IQGAP1 in breast cancer tissues

**Fig. 7:**
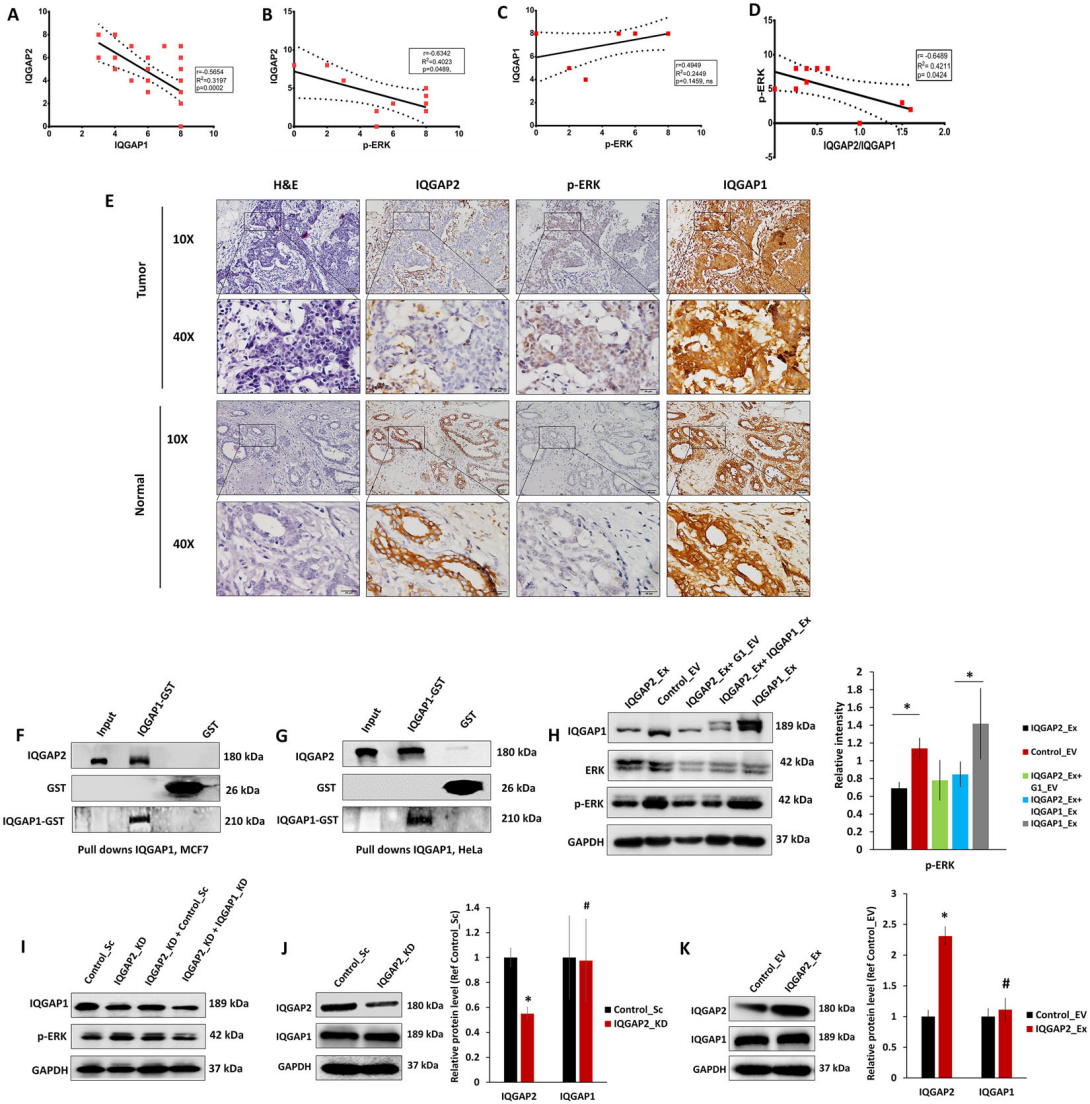
IQGAP2 is negatively correlated with phospho-ERK and interaction of IQGAP2 with IQGAP1 modulates IQGAP1 function. **A** Pearson correlation between the expression of IQGAP2 and IQGAP1 in tissues of breast cancer (*r* = −0.56, *n* = 38). **B** Pearson correlation between the expression of IQGAP2 and phospho-ERK (*r* = −0.63, *n* = 10) in tissues of breast cancer. **C** Pearson correlation between the expression of IQGAP1 and phospho-ERK (*r* = 0.49, *n* = 10) in tissues of breast cancer. **D** Pearson correlation between the expression of the ratio of IQGAP2/IQGAP1 and phospho-ERK (*r* = −0.64, *n* = 10) in tissues of breast cancer. In **A**–**D** solid and dashed line show regression line and standard error, respectively. **E** Representative IHC images of IQGAP2, phospho-ERK, and IQGAP1 in the normal and tumor region of a breast cancer patient. **F** Western blot images showing status of IQGAP2 in pull-down assay performed with IQGAP1_GST and GST only in MCF7 cells. The blots were probed with anti-IQGAP1, anti-IQGAP2, and GST antibodies. **G** Western blot images showing status of IQGAP2 in pull-down assay performed with IQGAP1_GST and GST only in Hela cells. The blots were probed with anti-IQGAP1, anti-IQGAP2, and GST antibodies. **H** Left panel shows Western blot of phospho-ERK in MCF7 cell having IQGAP2 overexpression (IQGAP2_Ex), IQGAP2 empty vector (Control_EV), IQGAP2 overexpression with IQGAP1 empty vector (IQGAP2_Ex + G1_EV), overexpression of IQGAP2 on IQGAP1 overexpression (IQGAP2_Ex + IQGAP1_Ex) background, and IQGAP1 overexpression (IQGAP1_Ex). Right panel shows corresponding densitometry data (*n* = 3). **I** Western blot of phospho-ERK in MCF7 cell having depletion of IQGAP1 and IQGAP2 (IQGAP2_KD + IQGAP1_KD), depletion of IQGAP2 with control vector for IQGAP1_KD (IQGAP2_KD + Control_Sc), IQGAP2 depletion (IQGAP2_KD), and its control vector (Control_Sc). **J** Western blot (left) showing expression of IQGAP1 in MCF7 with IQGAP2 reduction (IQGAP2_KD) and its control (Control_Sc). The bar graph (right panel) shows corresponding densitometry data (*n* = 3). **K** Western blot (left) image for IQGAP1 expression in MCF7 with IQGAP2 overexpression (IQGAP2_Ex) and its control (Control_EV). The bar graph (right panel) shows corresponding densitometry data (*n* = 3). Data are presented as mean ± SEM. Student’s *t* test, two-tailed unpaired was used for the comparison of means. ^#^*p* > 0.05, *p ≤ 0.05.

The reciprocal expression pattern of IQGAP1 and IQGAP2 in HCC^11,21^ prompted us to investigate the correlation between the ratios of these IQGAP isoforms in breast cancer. We analyzed Breast Invasive Carcinoma, TCGA, Firehose Legacy data and observed a negative correlation between MAPK1_PY187 and IQGAP2 (Supplementary Fig. 6A), whereas a non-significant but positive correlation was observed between MAPK1_PY187 and IQGAP1 (Supplementary Fig. 6B). As corroboration, we observed a strong negative correlation between IQGAP1 and IQGAP2 in breast cancer tissues (Fig. 7A).

Next, we examined the correlation between IQGAP2/phospho-ERK and IQGAP1/phospho-ERK in breast cancer tissues. A strong negative correlation between IQGAP2/phospho-ERK (Fig. 7B) and a positive but non-significant correlation between IQGAP1/phospho-ERK (Fig. 7C) were observed in breast cancer patients. Interestingly, there was a strong negative correlation between IQGAP2/IQGAP1 ratio and phospho-ERK (Fig. 7D). Representative IHC images are shown in Fig. 7E. These data together indicate that the ratio of IQGAP2 to IQGAP1 regulates the phospho-ERK level in breast cancer.

### IQGAP2 interacts with IQGAP1 and modulates IQGAP1-mediated ERK activation

Next, we sought to determine the possibility of a physical interaction between IQGAP1 and IQGAP2, which can lead to a sequestering effect on IQGAP1-mediated ERK activation. A clear IQGAP2 band was detected in pull downs with IQGAP1_GST using MCF7-IQGAP2-Ex cell lysate (Fig. 7F). Hela cell lysates also showed similar results (Fig. 7G), confirming an interaction between IQGAP1 and IQGAP2. Next, we tested whether the ratio of IQGAP2 to IQGAP1 has a functional effect on ERK activation. We found IQGAP1 ectopic expression activates ERK, and when we increased the level of IQGAP2 in MCF7 cells having high ectopic IQGAP1 expression, the phospho-ERK level reduced (Fig. 7H). Double knockdown of IQGAP1 and IQGAP2 counterbalanced the effect to IQGAP2 reduction on phospho-ERK (Fig. 7I), and on the migratory and proliferative properties of MCF7 (Supplementary Fig. 7A, B), which further confirms the effect of IQGAP1/IQGAP2 ratio on ERK activation. Change in IQGAP2 expression did not change IQGAP1 level which rules out the possibility of IQGAP2-mediated downregulation of IQGAP1 expression (Fig. 7J, K). We did not find interaction of KRAS with IQGAP1, but it showed binding with ERK, which increased with IQGAP2 depletion (Supplementary Fig. 8A–C). Perturbation of IQGAP2 level did not have any effect on KRAS level (Supplementary Fig. 8D, E). Therefore, we inferred that IQGAP2 level in the cells can modulate IQGAP1-mediated ERK activation.

## Discussion

The search for molecular markers, which could assist in early diagnosis of breast cancer irrespective of the molecular subtype, will aid in the development of an effective treatment regime against drug resistance. In light of the crucial roles played by other members of the IQGAP family across breast cancers molecular subtypes, we decided to explore the role of IQGAP2 in breast cancer progression.

Our IHC data revealed that IQGAP2 protein levels are significantly reduced in a large proportion of breast cancer cases, suggesting it to be a tumor suppressor, which is consistent with our previous datamining-based findings^19^. Although we did not find a statistically significant association between tumor size and IQGAP2 expression in patients, our in vitro and in vivo (xenograft model) results clearly showed the anti-proliferative role of IQGAP2 in breast cancer, which is consistent with the prior findings of IQGAP2 in other cancer types^9–12^. Deregulated cell proliferation and apoptosis acquired by the tumor cells result in continuous tumor growth and development of breast cancer^30^. In search of the mechanism, we could establish that IQGAP2 promotes apoptosis irrespective of the molecular subtype of breast cancer cells by activating the ROS-P38 pathway. This finding is in agreement with the observation of IQGAP2 and ROS level in HCC^5^. In our study, the loss of IQGAP2 expression resulted in increased IL-6 and CCL2 levels which have been reported to induce signaling through the MAPK pathway^31,32^. The above evidences substantiate the role of IQGAP2 in suppression of cell growth.

Lymph node metastasis is an early event of distant metastasis of tumor cells and an important indicator of prognosis for breast cancer patients^33^. Lymphovascular invasion is another independent prognostic factor, which precedes lymph node metastasis^34^. Our patient data showed an association of reduced IQGAP2 expression with higher lymph node metastasis and lymphovascular invasion, which is a testimony to the crucial tumor-suppressing ability of this protein. This was further substantiated by the association of reduced IQGAP2 expression with higher stages (III–IV). These results are in agreement with the previous reports of IQGAP2 in other cancer types^35,36^. IQGAP2 has been reported to promote the migration and invasion of HCC, prostate, ovary, and gastric cancer cells^9–12^. We also found that the knockdown of IQGAP2 promoted migration and invasion ability of breast cancer cells which was also evident in E-cadherin downregulation, and N-cadherin, Snail, Twist upregulation. Cytokines and chemokines like IL-6, IL-8, CCL2, CCL3, and CCL11 are important for maintaining aggressive traits in less invasive luminal cells as well invasive basal like cells^31,37–39^. We observed increased expression of these cytokines with reduced IQGAP2 expression, and supporting our observation on EMT.

Two major pathways, MAPK and AKT, promote migration and invasion in breast cancer^40^. Previous study has shown the role of AKT pathways in IQGAP2-mediated regulation of EMT in prostate cancer^10^. In our study, we observed the activation of MEK-ERK signaling leading to EMT with the loss of IQGAP2 expression. We found that IQGAP2 depletion in breast cancer lines did not affect AKT activation. Phosphorylated AKT has been shown to be a negative regulator of IQGAP1-mediated ERK activation in prostate cancer cells^41^. If the depletion of IQGAP2 had caused activation of AKT activity, it would have meant IQGAP1 dependent depletion of phospho-ERK, which we did not observe. Thus, our findings of IQGAP2-mediated depletion of total phospho-ERK levels, and no effect on activation of AKT is in line with the observed function of IQGAP2 as a tumor suppressor.

Binding of IQGAP1 to ERα through IQ motif has been reported to result in enhanced ERα functions^16^. In our study, the interaction of IQGAP2 with IQGAP1 and reduction of ERα activity both were observed. Whether this direct or indirect binding of IQGAP2 to IQGAP1 is responsible for the loss of ERα activity in the cells needs further investigation.

Our findings with GST-Pull-down experiments show the presence of an IQGAP2-IQGAP1 complex in breast cancer cells, hinting at the possibility of masking the pro-oncogenic effects of IQGAP1 and subsequent reduction of the phospho-ERK levels in the cells, similar to the phenomenon observed in HCC^21^. We saw a direct effect of IQGAP2 level on ERK binding, which explains increased phospho-ERK level, with decrease in IQGAP2. Role IQGAP1 in facilitating ERK activation is well known^42,43^. We did not observe the binding of KRAS with IQGAP1. Earlier studies have shown conflicting results regarding interaction of RAS with IQGAP1^44,45^. A recent study in a systematic analysis found that in breast cancer cells endogenous IQGAP1 did not bind to any of the RAS members, but if IQGAP1 expression level is ectopically increased, it binds to RAS^46^. Although our results suggest no binding of IQGAP1 with KRAS but due to experimental setup limitations we cannot completely rule out the possibility. More experiments are necessary to prove the significance of IQGAP1-IQGAP2 interaction with regards to breast cancer progression.

To summarize, we have established for the very first time the role of IQGAP2 as a tumor suppressor in breast cancer, which regulates MEK-ERK and p38 pathways to reduce the cancerous properties of cells. The ratio of IQGAP2/IQGAP1 would be crucial in assigning the prognostic significance for breast cancer.

## Supplementary information

Supplementary Figures

Supplementary Table 1

Supplementary Table 2

Supplementary methods

## Data Availability

All data generated or analyzed during this study are included in this published article and its Supplementary information files.
