# Supplementary material for: Reduced IQGAP2 expression promotes EMT and inhibits apoptosis by modulating the MEK-ERK and p38 signaling in breast cancer irrespective of ER status
Source: Cell Death Dis. 2021 Apr 12;12(4):389. doi: 10.1038/s41419-021-03673-0 (PMC8041781; doi:10.1038/s41419-021-03673-0)
Supplement: Supplementary file 1 — Supplementary Figures [file 41419_2021_3673_MOESM1_ESM.pdf]

SUPPLEMENTARY FIGURE

Supplementary Figure 1.

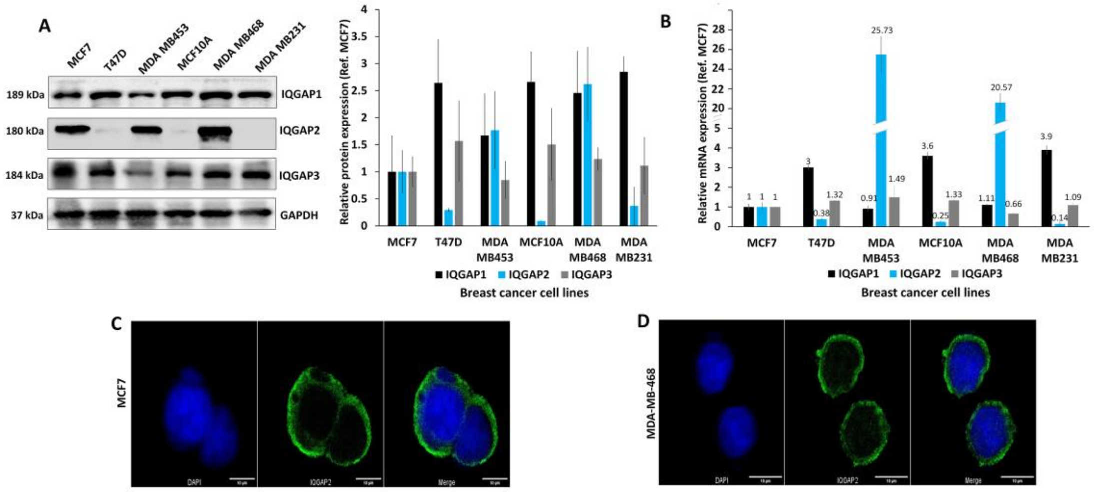

Supplementary Fig. 1: IQGAP2 expression doesn't correlate with breast cancer molecular subtype. A The Western blot of IQGAP1, IQGAP2 and IQGAP3 showing their endogenous expression in different breast cancer cell lines of specific molecular subtype (left panel). Here, expression is shown in MCF7 (ER/PR positive), T-47D (ER/PR/Her2 positive), MDA-MB-453 (Her2 positive), MCF 10A (ER/PR/Her2 negative normal like), MDA-MB-468 (ER/PR/Her2 negative) and MDA-MB-231 (ER/PR/Her2 negative and highly aggressive) cell lines. Right panel shows densitometry bar graph relative to the expression of MCF7, (n = 3). **B** The endogenous mRNA level of IQGAP1, IQGAP2 and IQGAP3 (relative to the endogenous expression in MCF7) in different breast cancer cell lines (n = 3). **C** The subcellular localisation of IQGAP2 in MCF7 depicted by immunofluorescence (AF-488) staining (n = 3). DAPI (blue) staining shows nucleus. **D** The subcellular localisation of IQGAP2 in MDA-MB-468 visualised by immunofluorescence (AF-488) staining (n = 3). The scale bar is 10 µm. Images were taken with a 60X oil immersion objective lens in a confocal microscope.

Supplementary Figure 2.

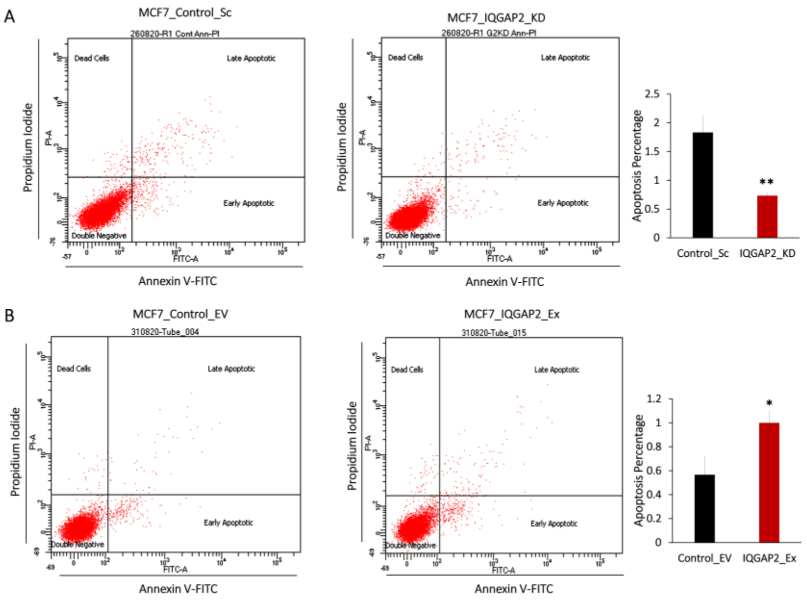

Supplementary Fig. 2: IQGAP2 expression levels affect apoptosis in breast cancer cell lines. Annexin V-FITC/Propidium Iodide based flow cytometry method was used to measure cell apoptosis. Percentage of apoptotic cells quantified by flow cytometry method depicts, **A** lower apoptosis in MCF7 cells with IQGAP2 depletion (MCF7_IQGAP2_KD) than control (MCF7_Control_Sc) group and, **B** higher apoptosis in MCF7 cells with IQGAP2 overexpression (MCF7_IQGAP2_Ex) than control (MCF7_Control_EV) group. Experiments were performed in triplicate and data is presented as mean ± S.E.M. Student's t-test, 2-tailed unpaired, was used for the comparison of means.* represents $p \leq 0.05$, ** represents $p \leq 0.01$.

Supplementary Figure 3.

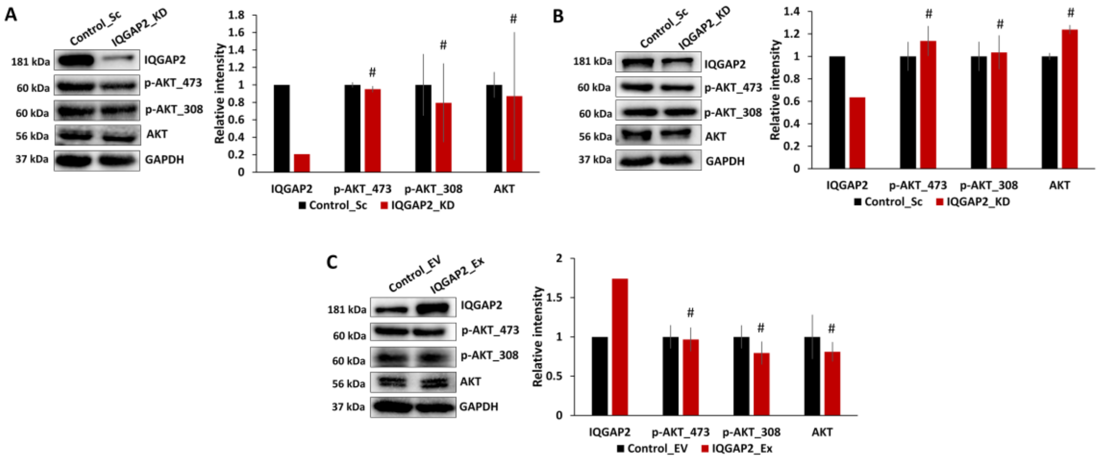

Supplementary Fig. 3: IQGAP2 expression level doesn't affect AKT activation. A Western blot showing no change in phospho-AKT_473 and phospho-AKT_308 level in MCF7 cells with IQGAP2 depletion (IQGAP2_KD) compared to control (Control_Sc). **B** Western blot showed no change in phospho-AKT_473 and phospho-AKT_308 levels in MDA-MB-468 cells with IQGAP2 depletion (IQGAP2_KD) compared to control (Control_Sc). **C** Western blot showing no change in phospho-AKT_473 and phospho-AKT_308 level in MCF7 cells with IQGAP2 overexpression (IQGAP2_Ex) compared to respective control (Control_EV). Experiments were performed in triplicate and data is presented as mean ± S.E.M. # represents $p > 0.05$. Student's t-test, 2-tailed unpaired, was used for the comparison of means.

Supplementary Figure 4.

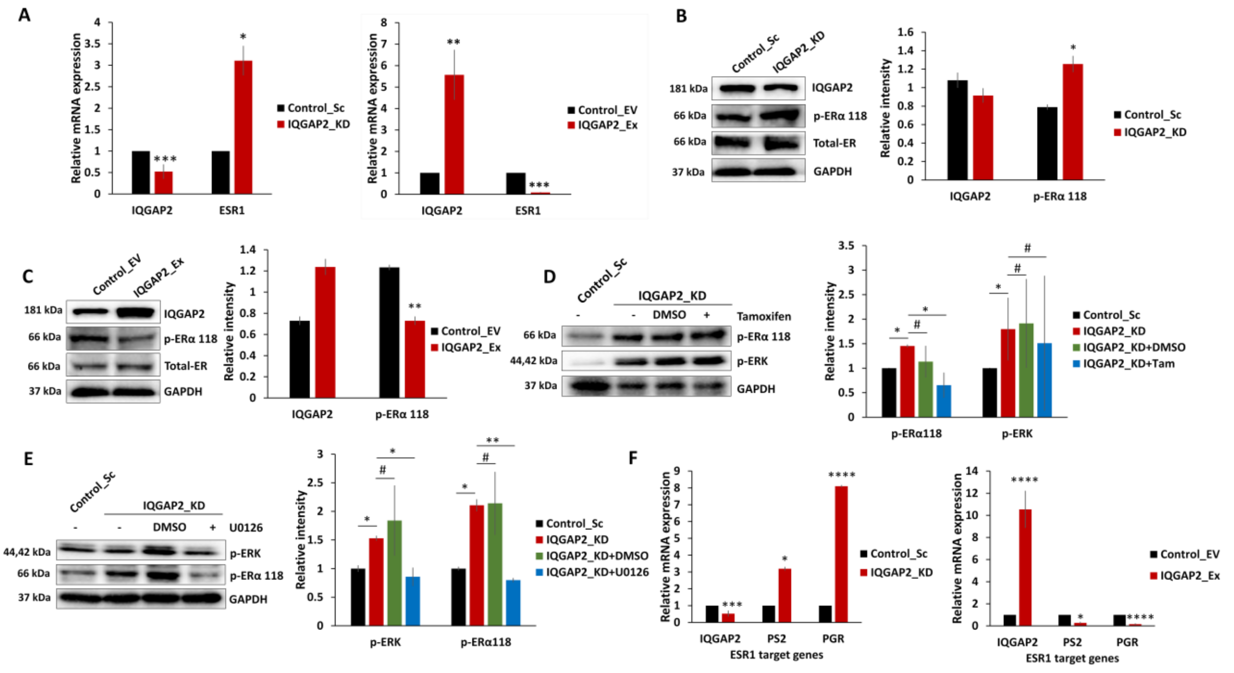

Supplementary Fig. 4: Reduction in IQGAP2 upregulates ER expression in MCF7 cells. **A** qRT-PCR data showing increased ESR1 mRNA expression in MCF7 cells with IQGAP2 depletion (IQGAP2_KD) compared to control (Control_Sc) (left panel). In the right panel, qRT-PCR data shows reduced ESR1 mRNA expression in MCF7 with IQGAP2 overexpression (IQGAP2_Ex) compared to the control (Control_EV). **B** Western blot showing increased phospho ERα^{ser118} level in MCF7 cells with IQGAP2 depletion (IQGAP2_KD) compared to control (Control_Sc) (left panel) and right panel shows corresponding densitometry bar graph. **C** Western blot showing reduced phospho ERα^{ser118} level in MCF7 with IQGAP2 overexpression (IQGAP2_Ex) compared to the control (Control_EV) (left panel) and right panel shows corresponding densitometry bar graph. **D** Western blot showing expression of phospho ERα^{ser118} and phospho-ERK in MCF7 cells with IQGAP2 depletion (IQGAP2_KD) and control (Control_Sc), after tamoxifen treatment (left panel). Right panel shows the corresponding densitometry bar graph. **E** Western blot showing expression of phospho-ERα^{ser118} and phospho-ERK in MCF7 cells with IQGAP2 depletion

(IQGAP2_KD) and control (Control_Sc), after treatment with ERK inhibitor II, U0126 (left panel). Right panel shows the corresponding densitometry bar graph. **F** qRT-PCR data showing PS2 and PGR mRNA expression in MCF7 cells with IQGAP2 depletion (IQGAP2_KD) and its control (Control_Sc) (left panel) and, in MCF7 with IQGAP2 overexpression (IQGAP2_Ex) and its control (Control_EV). Experiments were performed in triplicate and data is presented as mean ± S.E.M. Student's t-test, 2-tailed unpaired, was used for the comparison of means. # represents $p > 0.05$, * represents $p \leq 0.05$, ** represents $p \leq 0.01$, *** represents $p \leq 0.001$, **** represents $p \leq 0.0001$.

Supplementary Figure 5.

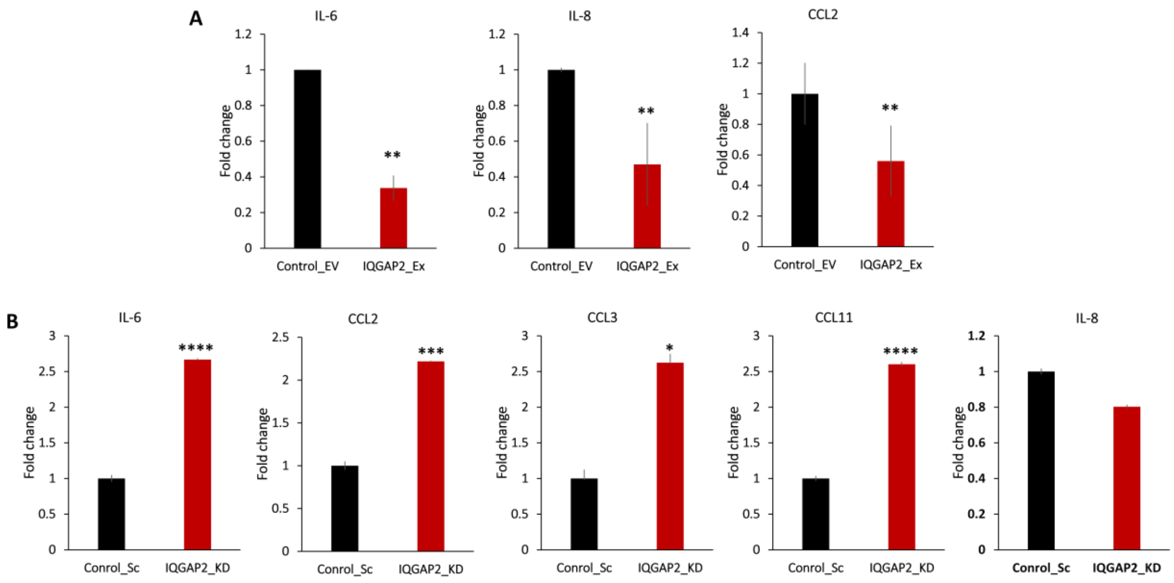

Supplementary Fig. 5: IQGAP2 expression reduces the pro-inflammatory cytokine expression in breast cancer cells. A Bar graph showing the differential transcript level of IL-6, IL-8 and CCL2 in MCF7_IQGAP2_Ex and MCF7_Control_EV groups. **B** Bar graph showing the differential transcript levels of IL-6, CCL2, CCL3, CCL11 and IL-8 between MDA-MB-468_IQGAP2_KD and MDA-MB-468_Control_Sc groups. Experiments were performed in triplicate and data is presented as mean ± S.E.M. Student's t-test, 2-tailed unpaired, was used for the comparison of means. * represents $p \leq 0.05$, ** represents $p \leq 0.01$, *** represents $p \leq 0.001$, **** represents $p \leq 0.0001$.

Supplementary Figure 6.

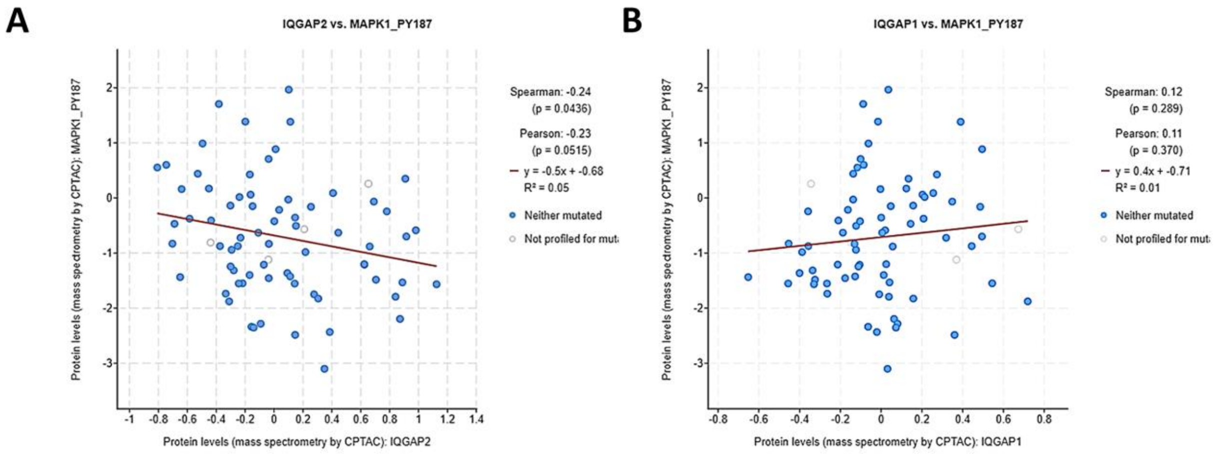

Supplementary Fig. 6: IQGAP2 negatively correlates with phospho-ERK level in TCGA, Firehose Legacy, cBioPortal dataset. A Graph showing a negative correlation between IQGAP2 and phospho-ERK (MAPK1_PY187) (r = -0.23, n = 74, Pearson correlation). **B** Graph showing a positive but non-significant correlation between IQGAP1 and phospho-ERK (MAPK1_PY187) (r = 0.11, n = 74, Pearson correlation).

Supplementary Figure 7.

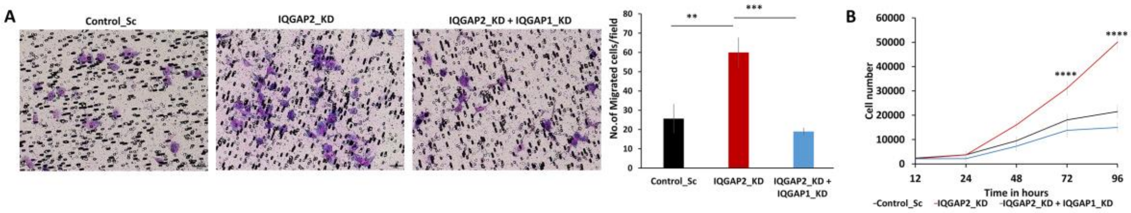

Supplementary Fig. 7: Effect of IQGAP1/IQGAP2 double knock down on migration and proliferation. A Left panel shows the transwell migration of MCF7 cells having depletion of IQGAP1 and IQGAP2 (IQGAP2_KD + IQGAP1_KD), depletion of IQGAP2 (IQGAP2_KD), and its control vector (Control_Sc). Right panel shows the bar graph for corresponding data for no. of migrated cells. **B** The graph shows cell proliferation (MTS assay) of MCF7 having reduction of IQGAP1 and IQGAP2 (IQGAP2_KD + IQGAP1_KD), reduction of IQGAP2 (IQGAP2_KD), and its control vector (Control_Sc). Experiments were performed in triplicate and data were presented as mean ± S.E.M. Student's t-test, 2-tailed unpaired, was used for the comparison of means. ** represents $p \leq 0.01$, *** represents $p \leq 0.001$, **** represents $p \leq 0.0001$.

Supplementary Figure 8.

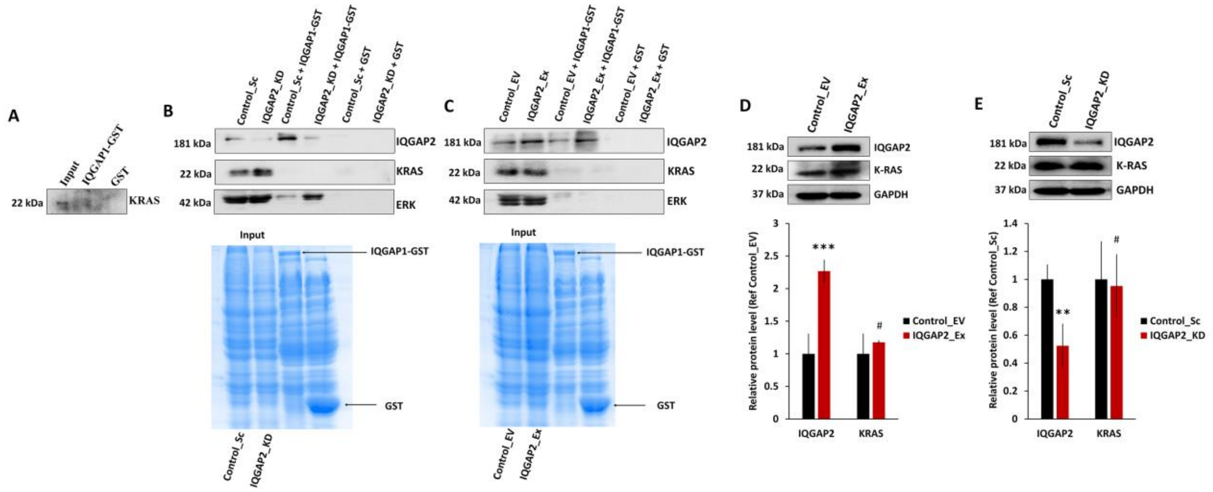

Supplementary Fig. 8: IQGAP2 depletion increases the binding of IQGAP1 and ERK but doesn't affect KRAS. A Western blot image shows binding status of KRAS with IQGAP1. Pull down assay was performed with IQGAP1_GST or GST only, in HeLa cells. The blot was probed with anti KRAS antibody. **B** Western blot images show binding status of IQGAP2, KRAS and ERK with IQGAP1 in IQGAP2 depletion and control conditions. Proteins were pulled using IQGAP1_GST or GST only, from MCF7 cells with IQGAP2 depletion (IQGAP2_KD) and its corresponding control (Control_Sc). **C** Western blot image show binding status of IQGAP2, KRAS and ERK with IQGAP1 in IQGAP2 over expression and control conditions. Proteins were pulled using IQGAP1_GST or GST only, from MCF7 cells with IQGAP2 over-expression (IQGAP2_Ex) and its corresponding control (Control_EV). **B-C** The blots were probed with anti-IQGAP2, KRAS and ERK antibodies (Upper panel). First two lanes show input and last four lanes show pulldown products. Lower panel shows Coomassie protein gel for inputs and, IQGAP1-GST and GST lysates. **D-E** D and E, Western blot images show the expression level of KRAS upon increased IQGAP2 level (IQGAP2_Ex) and upon IQGAP2 reduction (IQGAP2_KD) in MCF7 cells,

respectively. Right panel shows corresponding densitometry data ($n = 3$). Data is presented as mean ± S.E.M. Student's t-test, 2-tailed unpaired, was used for the comparison of means. # represents $p > 0.05$, ** represents $p \leq 0.01$, *** represents $p \leq 0.001$.
