# Supplementary material for: Reduced IQGAP2 expression promotes EMT and inhibits apoptosis by modulating the MEK-ERK and p38 signaling in breast cancer irrespective of ER status
Source: Cell Death Dis. 2021 Apr 12;12(4):389. doi: 10.1038/s41419-021-03673-0 (PMC8041781; doi:10.1038/s41419-021-03673-0)
Supplement: Supplementary file 2 — Supplementary Table 1 [file 41419_2021_3673_MOESM2_ESM.docx]

**Supplementary Table 1. List of qRT-PCR primers**

| **S.No.** | **Primer Name** | **Primer sequences (5' - 3')** |
| --- | --- | --- |
| **1** | IQGAP2 F | TTCAGTCCTGGTTCCGAATGGC |
| **2** | IQGAP2 R | TGTTCGCTCTCAACAGTGACTGT |
| **3** | GAPDH F | ACCCAGAAGACTGTGGATGG |
| **4** | GAPDH R | TCTAGACGGCAGGTCAGGTC |
| **5** | CCL11 F | ACCCCTTCAGCGACTAGAGA |
| **6** | CCL11 R | CTTGAAGATCACAGCTTTCTGGG |
| **7** | CCL3 F | TTCCGTCACCTGCTCAGAAT |
| **8** | CCL3 R | GCAGCAAGTGATGCAGAGAAC |
| **9** | CCL2 F | GCTCAGCCAGATGCAATCAAT |
| **10** | CCL2 R | CACTTGCTGCTGGTGATTCTT |
| **11** | IL6 F | TGCGTTGCACTTGTTTACGC |
| **12** | IL6 R | GGAGGCTACCACTTCCACCT |
| **13** | IL8 F | ACCGGAAGGAACCATCTCAC |
| **14** | IL8 R | GGCAAAACTGCACCTTCACAC |
| **15** | PGR F | GTGCCTATCCTGCCTCTCAATC |
| **16** | PGR R | CCCGCCGTCGTAACTTTCG |
| **17** | PS2 F | CATCGACGTCCCTCCAGAAGAG |
| **18** | PS2 R | CTCTGGGACTAATCACCGTGCTG |
| **19** | ESR1 F | GCTACGAAGTGGGAATGATGAAAG |
| **20** | ESR1 R | TCTGGCGCTTGTGTTTCAAC |
| **21** | IQGAP3 F | GTTCATCCATAGAGCCTGCCA |
| **22** | IQGAP3 R | GCGATGCTCTCACCAATAAGG |
| **23** | IQGAP1 F | GATGAGCTGCTGAAGATTATTGGT |
| **24** | IQGAP1 R | ATGTCAAAGGCATCAGGAGCA |

**Abbreviations:** F-Forward primer sequence, R-Reverse primer sequence
