# Supplementary material for: Reduced IQGAP2 expression promotes EMT and inhibits apoptosis by modulating the MEK-ERK and p38 signaling in breast cancer irrespective of ER status
Source: Cell Death Dis. 2021 Apr 12;12(4):389. doi: 10.1038/s41419-021-03673-0 (PMC8041781; doi:10.1038/s41419-021-03673-0)
Supplement: Supplementary file 3 — Supplementary Table 2 [file 41419_2021_3673_MOESM3_ESM.docx]

**Supplementary Table 2.** List of primary antibodies used in Western blot

| **S.No.** | **Name of antibody** | **Dilution** | **Brand** |
| --- | --- | --- | --- |
| 1 | Anti- IQGAP2 | 1:1000 | Abcam, MA, USA |
| 2 | Anti- IQGAP1 | 1:1000 | Abcam, MA, USA |
| 3 | Anti-N-cadherin | 1:1000 | Abcam, MA, USA |
| 4 | Anti-E-cadherin | 1:1000 | Abcam, MA, USA |
| 5 | Anti-Twist | 1:1000 | CST, Inc., MA, USA |
| 6 | Anti-Snail | 1:1000 | CST, Inc., MA, USA |
| 7 | Anti-AKT | 1:1000 | CST, Inc., MA, USA |
| 8 | Anti-phospho-AKT473 | 1:1000 | CST, Inc., MA, USA |
| 9 | Anti-phospho-AKT308 | 1:1000 | CST, Inc., MA, USA |
| 10 | Anti-ERK | 1:1000 | CST, Inc., MA, USA |
| 11 | Anti-phospho-ERK | 1:1000 | CST, Inc., MA, USA |
| 12 | Anti-P38 | 1:1000 | CST, Inc., MA, USA |
| 13 | Anti-phospho-P38 | 1:1000 | CST, Inc., MA, USA |
| 14 | Anti-GST | 1:1000 | CST, Inc., MA, USA |
| 15 | Anti-GAPDH | 1:10000 | Sigma, Missouri, USA |
