# Supplementary material for: Reduced IQGAP2 expression promotes EMT and inhibits apoptosis by modulating the MEK-ERK and p38 signaling in breast cancer irrespective of ER status
Source: Cell Death Dis. 2021 Apr 12;12(4):389. doi: 10.1038/s41419-021-03673-0 (PMC8041781; doi:10.1038/s41419-021-03673-0)
Supplement: Supplementary file 4 — Supplementary methods [file 41419_2021_3673_MOESM4_ESM.docx]

**Supplementary methods**

**Quantitative real-time PCR**

Total RNA was isolated using RNeasy Mini Kit (Qiagen, Hilden, Germany) as per the manufacturer’s protocol. 1 µg of RNA was converted into cDNA using Verso cDNA synthesis kit (Thermo Scientific). The primer sequences for genes screened in this study have been shown in the Supplementary Table 1. Real-time PCR was performed in QuantStudio™ 7 Flex Real-Time PCR System (Thermo Scientific), using PowerUp™ SYBR® Green Master Mix (Thermo Scientific). GAPDH was used as internal control. 2^−∆∆CT^ method was used to calculate the relative expression of gene.

**Western blot analysis**

Cells were lysed in RIPA buffer (Thermo Scientific), and protease-phosphatase inhibitor (Sigma) was added. 20 µg of total protein was immunoblotted using appropriate primary (Supplementary Table 2) and secondary antibodies followed by detection using SuperSignal™ West Femto reagent (Thermo Scientific). The chemiluminescence signals were detected in ChemiDoc XRS+ (Bio-Rad, CA, USA).

**Cell proliferation assay**

Cell proliferation assay was performed using MTS CellTiter 96® AQueous One Solution Reagent (Promega, WI, USA) as per the manufacturer’s protocol. The final absorbance was taken at 495nm using Varioskan Flash multimode reader (Thermo Scientific).

**Colony formation assay**

Cells were seeded into 6-well plate at a density of 1000 cells/well. After incubation, the cells were washed, fixed with methanol and acetic acid (3:1) solution for 5 min and stained with 0.5% crystal violet (MP Biomedicals, Valiant, China). Cell colonies were photographed by digital camera (Nikon, Tokyo, Japan) and were counted using ImageJ software (NIH, MD, USA).

**Wound healing assay**

Cells (0.2 × 10^6^) from each experimental group were seeded in 12-well plates. The monolayer was scraped with a 200 µl pipette tip. Images of wound were captured every 6 h till 24 h. The area of wound was calculated using ImageJ software.

**Transwell cell migration and invasion assay**

For transwell migration assay, 0.05 × 10^6^ cells were seeded in 500 µl of reduced serum medium in the upper chamber of a 12-well plate. 1 ml of complete DMEM was filled in the lower chamber and incubated for 24 h. Cells were fixed with 4% paraformaldehyde and stained with 0.5% crystal violet (HiMedia). Cells from the upper chamber were wiped off and images were captured under microscope (Olympus, Tokyo, Japan). The counting of cells was done using ImageJ software.

For transwell invasion assay, in the initial step transwell chambers were coated with 100 μl of 1 mg/ml growth factor reduced Matrigel (Corning, NY, USA).
